# A Path Model for Subjective Well-Being during the Second Wave of the COVID-19 Pandemic: A Comparative Study among Polish and Ukrainian University Students

**DOI:** 10.3390/jcm11164726

**Published:** 2022-08-12

**Authors:** Aleksandra M. Rogowska, Cezary Kuśnierz, Iuliia Pavlova, Karolina Chilicka

**Affiliations:** 1 Institute of Psychology, University of Opole, 45-052 Opole, Poland; 2 Faculty of Physical Education and Physiotherapy, Opole University of Technology, 45-758 Opole, Poland; 3 Department of Theory and Methods of Physical Culture, Lviv State University of Physical Culture, 79007 Lviv, Ukraine; 4 Department of Health Sciences, University of Opole, 45-040 Opole, Poland

**Keywords:** anxiety, coronavirus-related PTSD, depression, gender, mental health, perceived stress, physical health, relationship status, university students, well-being

## Abstract

Background: Previous studies showed several associations between physical and mental health dimensions and well-being. This study aims to examine a complex path model explaining the life satisfaction of university students from Poland and Ukraine during the second wave of the COVID-19 pandemic. Methods: The cross-sectional web-based study was performed in November 2020 using Google Forms. The conventional sample of 3230 university students from Poland (*n* = 1581) and Ukraine (*n* = 1649), aged 18–59 (*M* = 21.40, *SD* = 3.46), with 59% women, participated in the study. We used standardized questionnaires to measure life satisfaction (SWLS), self-reported physical health (GSRH), perceived stress (PSS-10), coronavirus-related PTSD (PCL-S), anxiety (GAD-7), and depression (PHQ-9). We also developed some questions to assess the exposure to the COVID-19 pandemic, positive effects of the pandemic, religiosity, and physical activity (PA). Results: We found a high prevalence of stress, coronavirus-related PTSD, anxiety, and depression and a low level of life satisfaction and physical health. Polish students, women, and those with insufficient PA levels reported worse physical and mental health than Ukrainians, men, and those who exercised sufficiently during the pandemic. Low perceived stress can directly predict life satisfaction, anxiety, and depression. Low stress also leads to better physical health, sufficient PA levels, high religiosity, and more perceived positive effects of the pandemic. Several indirect effects between particular variables and life satisfaction were also found in the path model. Conclusions: The target group for campus prevention programs is Polish university students, women, and people with insufficient PA levels. Intervention and prevention programs should focus on coping strategies and techniques for improving mental and physical health.

## 1. Introduction

The COVID-19 pandemic impacted the physical and mental health of populations worldwide. University and college students were especially vulnerable [1,2,3,4,5,6,7,8]. Many restrictions were implemented to prevent coronavirus spread during the lockdown, which forced distance work and education and limited access to sports and physical activity, increasing stress and anxiety among students [9,10]. University and college students reported concerns about online exams and classes or the continuation of education, their mental and physical health deterioration, poorer social relationships with peers and family members, financial status, and employment stability [11,12,13]. Brooks et al. [14] suggested that the adverse consequences of lockdown can affect mentally and physically quarantined individuals and the whole healthcare system. Meanwhile, the pandemic has not only shown a negative impact on human life but plenty of positive effects of the lockdown were reported among people around the world, including spending more time with other people, improved communication and greater investment in family life, more free time for entertainment and relaxation, development of interests and hobbies, and increased creativity and technological innovation [15,16,17,18].

In November 2020, the number of new cases of coronavirus infection in Ukraine increased rapidly. At the beginning of November, about 9000 cases were diagnosed daily; on 26 November, the number of cases exceeded 15,000. Due to this, several restrictions were implemented across the country. In particular in November, the so-called weekend quarantine was implemented. According to this, public catering facilities, shopping centers, cinemas, gyms, fitness centers, swimming pools, cultural institutions, etc., were closed on Saturdays and Sundays. Restrictions were also introduced on weekdays. In particular, it was announced that such limits, previously implemented in regions of the country with high morbidity in the previous months (in the so-called orange zones), would apply to the whole country. Among such restrictions was a prohibition on mass sports and religious events with more than 20 participants, the operation of cinemas at more than 50% capacity, all hostels, public transport with more people than the number of seats, no more than one person per 20 m^2^ of space in gyms and fitness centers, entrance and movement in restaurants restricted to those wearing masks, and restrictions on the distance between tables in restaurants and cafes, the number of people at a table, prohibition of operation at night, etc. Furthermore, the organization of activities of higher education institutions, in particular distance learning, was assigned to the appropriate management body of the educational institution. At the same time in November, the Ministry of Education and Science of Ukraine recommended a prohibition of visits to educational institutions and study groups of more than 20 people. The ministry recommended conducting studies in a mixed form (distance learning and, if possible, face-to-face consultations and small group seminars) given the epidemic situation in a specific region and educational institution.

Similar restrictions were introduced in November 2020 in Poland. Masks were mandated in public spaces (e.g., in shops, buses, and streets) throughout the country from 10 October. The other restrictions included a limit of one person per 4 m^2^ for events such as fairs, exhibitions, congresses, or conferences; a limit of one person per 7 m^2^ in gyms; a limit of 100 people, excluding service personnel, at family events; and a limit of 50% capacity in churches or other places of worship. The Polish government announced additional restrictions effective from 7 November until 29 November 2020. Remote learning in schools was mandated. All cultural institutions and shops except grocery stores, chemists, hardware stores, pet stores, and newsstands were closed. Occupancy in retail outlets up to 100 m^2^ was limited to one person per 10 m^2^; occupancy in churches was limited to one person per 15 m^2^, and hotels could only accept guests traveling for work purposes. Previous restrictions were also extended until 29 November 2020. Due to the growing number of infections and concern for the safety of students and employees of the Opole University of Technology and the University of Opole, all classes were conducted remotely from 1 November until the end of 2020.

Systematic reviews, meta-analyses, and longitudinal data showed that stress, anxiety, and depression increased significantly compared to the pre-pandemic period, deteriorating well-being (including life satisfaction) successively during the following waves of the COVID-19 pandemic [6,19,20,21,22,23,24,25]. For example, Fruehwirth et al. [22] showed in a longitudinal study that the prevalence of moderate–severe anxiety increased from 18.1% to 25.3%, while the prevalence of moderate–severe depression increased from 21.5% to 31.7% during the first month of the pandemic among first-year university students from the USA. Furthermore, female students were at the highest risk of increased anxiety and depression symptoms. Apart from females [3,5,6,26,27,28,29,30,31,32,33,34,35,36], young age [4,28,29,30,37,38,39,40,41,42,43,44] and single relationship status also [3,30] increased the risk of mental health issues during the pandemic.

It is important to note that the prevalence of stress, anxiety, and depression is dependent on the geographic region [3,23]. As evidenced in previous studies, university students and young adults from various countries have displayed different mean levels and associations between particular variables related to mental health and well-being during the COVID-19 pandemic, including life satisfaction, self-rated physical health, stress, coronavirus-related PTSD, fear of vaccination, fear of COVID-19, anxiety, and depression during the first and second waves of the pandemic [4,5,30,31,45,46,47]. In contrast, country (Germany, Israel, Poland, and Slovenia), sex, and age did not moderate the association of coronavirus-related PTSD with perceived stress, fear of COVID-19, fear of vaccination, and trust in institutions in a recent study among young adults during the third wave of the COVID-19 pandemic [48]. More research is required to examine whether age, gender, or country are associated with various dimensions of well-being in different timelines of the pandemic.

The cross-cultural differences in mental health and well-being during the pandemic may be related to various levels of restrictions in particular countries during the pandemic and also to the individual level of exposure to COVID-19 [2,7,9,46]. The study showed that exposure to COVID-19 increased significantly from the first to the second wave of the pandemic among university students from six countries (including Poland and Ukraine) [46]. Considering various categories of exposure to COVID-19, the most important predictors of coronavirus-related PTSD (cut-off score in PCL was 44) were the previous diagnosis of depression, death of friends or relatives, job loss, and worsening economic status among students. Furthermore, religiosity was found to be a significant longitudinal predictor of a decrease in fear of COVID-19 [31]. Previous studies showed that religiosity can buffer the negative consequences of psychological distress, providing a sense of security, reducing fear and anxiety, and increasing well-being levels [49,50,51]. A recent meta-analysis of longitudinal studies confirmed that there is a consistent but small association between religiosity or spirituality and such mental health dimensions as distress, negative and positive mood, life satisfaction, and well-being [52].

Regular physical activity (PA) has a beneficial effect on the cardiovascular, muscular, and nervous systems, improving physical health and mental well-being [53,54,55,56]. Unfortunately, a recent study showed that PA levels decreased while sedentary behavior simultaneously increased during the first phases of the COVID-19 pandemic due to social distancing and lockdown [8,34,47,55,56,57,58,59,60,61,62,63,64]. Furthermore, research showed that negative changes in health-related behavior (including PA) were associated with more symptoms of depression, anxiety, and stress [60,65,66]. In addition, Rogowska et al. [63] found that physically active university students from Ukraine scored significantly lower in anxiety and depression than their inactive counterparts. A sufficient level of PA (minimum 150 min per last week) was found in 43% of the student sample [63]. Studies suggest that perceived stress is related positively to anxiety and depression [6,32,67] and inversely to life satisfaction [25,68]. In addition, physical and mental health were significant positive predictors of life satisfaction during the pandemic [69]. In contrast, high distress, anxiety, depression, and somatic symptoms are inversely related to subjective well-being [6,70,71,72]. The relationship between poor physical health with high stress, depression, and anxiety, as well as low subjective well-being, was found in previous studies [6,32,36,47,73].

### The Purpose of the Present Study

This study aims to find the complex associations between mental and physical health dimensions, to explain the interplay mechanism between particular variables and their impact on well-being in university students during the second wave of the COVID-19 pandemic. Summarizing the previous studies, high and prolonged stress during the pandemic can contribute to increased anxiety, depression, and PTSD and worsen physical health and life satisfaction. Well-being is inversely related to stress, anxiety, depression and PTSD, and poor health status. Perceived positive effects of the COVID-19 pandemic may increase subjective well-being. In addition, religiosity and PA level may predict better mental health and well-being. Moreover, such demographic variables as female gender and single relationship status can worsen mental health and well-being. Our previous research showed that selected associations are involved in several separate models of regression. In the present study, we will consider all variables in one complex model, which allows us to see all these variables from a better perspective and avoid bias related to intercorrelations and mutual influence of interactions between these variables. Furthermore, there is still limited information about cross-cultural differences in mental health during the COVID-19 pandemic. In particular, little is known about the mental health of university students from Ukraine.

According to the holistic biopsychosocial model of health and well-being [74,75], both health and disease are determined by a dynamic interplay between biological, psychological, and social factors. Any one of these factors alone is insufficient to explain health outcomes and subjective well-being (including life satisfaction) that are caused by interaction between biological factors (e.g., genetic predispositions for some somatic diseases and mental disorders, history of individual trauma or infection, current exposure to disease, biological sex, age), psychological factors (e.g., subjective sense of mental health and health-related behavior, personality traits, current level of perceived health status, stress, PTSD, anxiety, depression, religiosity, physical activity, perceived positive effects of the pandemic), and socio-cultural factors (socioeconomic status, gender, relationship status, religion, the culture of the country of origin, characteristic of environment depending on the geographical region or degree of urbanization, public health issues, or the political context). In the present study, the associations will be examined using the biopsychosocial model of health and well-being [76].

In addition, to better identify groups at risk for lower well-being levels, a sensitivity analysis will be performed, examining country, gender, age, relationship status, and PA levels in mental and physical health dimensions during the pandemic. The following hypotheses were formulated based on previous studies:

**Hypothesis** **1** **(H1).** *There is a high prevalence of stress, coronavirus-related PTSD, anxiety, and depression among university students during the second wave of the COVID-19 pandemic. In addition, participants frequently reported low life satisfaction levels and poor physical health during the pandemic.*

**Hypothesis** **2** **(H2).** *Polish and Ukrainian university students differ significantly in mean levels of mental and physical health*.

**Hypothesis** **3** **(H3).** *Women experience subjectively poorer physical health and well-being and higher levels of stress, coronavirus-related PTSD, anxiety, and depression compared to men and nonbinary people.*

**Hypothesis** **4** **(H4).** *People with insufficient levels of physical activity demonstrate poorer physical health and well-being and higher levels of stress, coronavirus-related PTSD, anxiety, and depression, compared to individuals exercising sufficiently (PA > 150 min per week).*

**Hypothesis** **5** **(H5).** *Satisfaction with life can be predicted by higher religiosity, more perceived positive effects of the pandemic, better physical health and sufficient PA levels, and low scores in perceived stress, coronavirus-related PTSD, anxiety, and depression, controlling for age, country, gender, and relationship status (as confounding variables).*

**Hypothesis** **6** **(H6).** *There are significant differences between Polish and Ukrainian university students in the complex path model for predictors of life satisfaction during the second wave of the COVID-19 pandemic.*

## 2. Materials and Methods

### 2.1. Study Design and Procedure

The cross-sectional study was performed in November 2020, during the second wave of the COVID-19 pandemic. The Ukrainian sample of university students participated in the study between 2 and 26 November, while the Polish group was recruited between 3 and 27 November. The survey was developed in Polish and English and then translated into Ukrainian and back-translated due to the guidelines of the translation and cross-cultural adaptation [77,78].

The invitation to the study was disseminated to groups related to universities (Lviv State University of Physical Culture, Opole University of Technology, and the University of Opole), using Facebook, Viber groups, and Telegram channels. In addition, during the online classes, university teachers encouraged their students to participate in the study, inviting them by sharing the link to the survey using Moodle and Teams online educational platforms. Participants from Poland were recruited from Opole University of Technology, and the University of Opole. Ukrainian students were recruited from Lviv State University of Physical Culture. The studies were anonymous and voluntary.

The international study was performed consistent with the principles of the Declaration of Helsinki, and the study protocol was approved by the University Research Ethics Committee at the University of Opole (Decision No. 7, 29 October 2020), and by the Bioethics Committee of Lviv State University of Physical Culture (Decision No. 6, 16 December 2020). This study was preregistered on 7 October 2020, in the Open Science Framework (OSF) as a part of the international research project [79].

### 2.2. Measures

The online survey was prepared using Google Forms and consisted of several parts: (1) information about the study and informed consent; (2) demographic information; (3) exposure to the COVID-19 pandemic; (4) standardized questionnaires to measure religiosity, PA, life satisfaction, physical health, and mental health dimensions such as perceived stress, coronavirus-related PTSD, anxiety, and depression; and (5) perceived positive effects of the COVID-19 pandemic on life.

#### 2.2.1. Sociodemographic Survey

Students responded to several demographic questions about age (date of birth), gender (women, men, nonbinary), relationship status (single, in a couple), identification with a religious group (no religion, Muslim, Jewish, Catholic, Orthodox, Protestant, Methodist, Jehovah’s Witness, Buddhist, Hindu, other religion). According to the information about the study, the questions regarding the faculty (open question), field of study (open question), study level (bachelor, master, postgraduate, or doctoral), study grade (1–5 study year), and the type of study (full-time, part-time).

#### 2.2.2. Exposure to the COVID-19 Pandemic

The self-reported measure of exposure to the COVID-19 pandemic was developed for the study purpose in accordance with the methodology by Tang et al. [80] and used several times in previous studies [46,47,48,63,81]. Exposure to the COVID-19 pandemic was reported on eight questions. The participant replied whether or not (no = 0, yes = 1) he/she had each experience: (1) symptoms of COVID-19; (2) coronavirus tests; (3) hospitalization due to coronavirus infection; (4) isolation for at least 14 days due to infection; (5) infection with the coronavirus among family members or loved ones; (6) death from coronavirus of close relative; (7) job loss during the COVID-19 pandemic; and (8) deterioration in economic status during the pandemic.

#### 2.2.3. Positive Effects of the COVID-19 Pandemic

The positive effects of the COVID-19 pandemic were assessed by four questions developed by the authors for the study purpose. The question was: “I think that the situation associated with the coronavirus pandemic (COVID-19) has positively affected my life through (1) spending more quality time with family; (2) spending more quality time with friends; (3) spending more time relaxing and on entertainment; (4) spending more time on personal development and interests (hobby)”. Participants responded to each item on a 7-point Likert scale (from *Strongly disagree* = 1, to *Strongly agree* = 7). The reliability coefficient in the study sample was Cronbach’s α = 80.

#### 2.2.4. Religiosity

The measure of religiosity was developed on the basis of the Baylor Religion Survey [82] and was used in our previous research [31]. This is a single question “How religious do you consider yourself to be?”, to which the respondent answers on a four-point Likert scale (where *Not at all religious =* 0, while *Very religious =* 3).

#### 2.2.5. Physical Activity

Physical activity (PA) was measured using two questions about (1) the number of days per week when a person exercised, regarding the past month (with a 7-point response scale, from *Not one day* = 0, to *Seven days a week* = 7); and (2) the number of minutes (on average) of this physical activity per day (open question). The results of the two questions were multiplied to obtain the number of minutes of PA during the last week. The outcome was categorized as sufficient (PA ≥ 150 min per last week) or insufficient exercise level (PA < 150 min weekly), according to recommendations of the World Health Organization (WHO) [83]. The measure of PA was previously used in several studies [47,63,81,84].

#### 2.2.6. Perceived Physical Health

Health-related quality of life was assessed using the General Self-Rated Health (GSRH), developed by DeSalvo et al. [85,86]. The GSRH includes two items derived from the standard general health survey (SF-12V). A participant rates on a 5-point Likert scale (from *Excellent* = 1, to *Poor* = 5) his/her health individually (item 1) and in comparison to others same age (item 2). Higher scores are interpreted as worse perceived health. The reliability coefficient in the study was Cronbach’s α = 84.

#### 2.2.7. Life Satisfaction

The global cognitive aspect of subjective well-being was measured in the study using the Satisfaction With Life Scale (SWLS) developed by Diener et al. [87]. The SWLS consists of five items with a 7-point Likert scale of response (*Strongly disagree* = 1, while *Strongly agree* = 7). The scores range from 5–35, and a higher sense of satisfaction with life is represented by higher scores. The total scores can be categorized: extremely dissatisfied (5–9), dissatisfied (10–14), slightly dissatisfied (15–19), neutral (20), slightly satisfied (21–25), satisfied (26–30), and extremely satisfied (31–35). The internal consistency of the SWLS in the current study was Cronbach’s α = 0.83.

#### 2.2.8. Perceived Stress

The Perceived Stress Scale (PSS-10) was developed by Cohen et al. [88] for the assessment of whether a given life event is assessed as stressful. This is a short, 10-item scale with questions regarding the frequency of stressful situations in the past month. Participants rate their responses on a five-point Likert scale (from *Never* = 0, to *Very often* = 4). The total score is a sum of responses for each of the 10 items (ranging from 0–40), and higher scores indicate a higher stress level. The total score of perceived stress can be categorized as follows: extremely low (5–11), low (12–17), average (18–23), high (24–28), and extremely high (29–35). The internal consistency of the PSS-10 was Cronbach’s α = 0.86.

#### 2.2.9. Coronavirus-Related Post-Traumatic Stress Disorder

The coronavirus-related posttraumatic stress disorder (PTSD) was measured using the PTSD Check List—Specific (PCL-S) version developed by Weathers et al. [89,90] to assess specific stressful-event-related PTSD. The PCL-S comprises 17 items describing various stressful experiences. Participants respond how much they struggled with stress during the past month, using a five-point Likert scale (*Not at all* = 1, while *Extremely* = 5). All scores from 17 items are summarized into one composite score (ranging from 17–85), and a higher total result indicates a higher PTSD risk. We modified the PCL by adding to each item specification of the stressful event in regard to the COVID-19 pandemic and used the coronavirus-related PTSD in several previous studies [30,45,46,48]. An example of an item is: “Repeated, disturbing memories, thoughts, or images *of a* *stressful experience from the COVID-19 lockdown*”). The cut-off 44 was assumed in the study as coronavirus-related PTSD risk [91] to maximize diagnosis efficiency. The Cronbach’s α in the study was 0.92.

#### 2.2.10. Anxiety

Anxiety was assessed by the 7-item Generalized Anxiety Disorder (GAD-7) scale, developed by Spitzer et al. [92]. The GAD-7 is a short measure for a clinical assessment of anxiety risk during the last two weeks. Participants responded to each of seven items on how often they experienced anxiety symptoms in the past two weeks, using a 4-point Likert scale (*Not at all* = 0, *Nearly every day* = 3). Higher scores (ranging from 0–21) indicate higher anxiety. Scores can be categorized as no anxiety (0–4), mild (5–9), moderate (10–14), and severe (15–21) GAD risk. The Cronbach’s α in the present sample was 0.92.

#### 2.2.11. Depression

Depression was measured using the 9-item Patient Health Questionnaire (PHQ-9), developed by Kroenke et al. [93] to assess depression risk. Participants answer how frequently each symptom of depression occurred during the past two weeks, using the 4-point Likert response scale (*Not at all* = 0, *Nearly every day* = 3). The higher the score is (ranging from 0–27), the higher the depression risk. The total score can be categorized as no depression (0–4), mild (5–9), moderate (10–14), moderately severe (15–19), and severe (20–27) depression symptoms. The reliability coefficient in the present study was Cronbach’s α = 0.89.

### 2.3. Participants

Initially, 1713 Ukrainian students were invited to the study, but 64 refused to participate, so the final Ukrainian sample consisted of 1649 people (the response rate was 96.26%). Among Polish university students, 1699 responded to the invitation, while 118 did not agree to participate in the study. Therefore, the total sample included 1581 individuals (the response rate was 93.06%).

A total sample of 3230 university students participated in the study, including 1581 individuals from Poland (48.95%) and 1649 from Ukraine (51.05%). The mean age ranged from 18 to 59 (*M* = 21.40, *SD* = 3.46). Students represented various faculties of technical and humanities university types, including economy and political sciences (e.g., economy, human resources, management, law, politics, and social communication), engineering (architecture and construction, electrical engineering, energy, logistics, mechanical engineering, and transport technologies), health sciences (cosmetology, emergency, nursing, physical education, physiotherapy, tourism, and recreation), humanities (e.g., archeology, fine arts, culture, languages, and history), information technologies (e.g., informatics and computer sciences), social sciences (e.g., psychology, sociology, and pedagogy), and science (e.g., biology, chemistry, mathematic, physic, and geography). Most students were at the bachelor’s level (82.90%), and fewer were at the master’s (16.94%) and postgraduate or doctoral (0.16%) levels. Participants were in the following years of study: first (*n* = 1106, 34.24%), second (*n* = 1003, 31.05%), third (*n* = 613, 19.98%), fourth (*n* = 394, 12.20%), and fifth (*n* = 114, 3.53%). The majority of individuals were full-time students (*n* = 2964, 91.77%) rather than part-time (*n* = 266, 8.23%).

Most students identified as Catholic (*n* = 1259, 38.99%), Orthodox (*n* = 631, 19.54%), Greco-Catholic (*n* = 596, 18.46%), Protestant (*n* = 45, 1.39%), Buddhist (*n* = 19, 0.59%), Jewish (*n* = 1, 0.03)%, and another religion (*n* = 19, 0.59%), while 660 people did not identify with any religion (20.43%). Regarding gender, most students were women (59.20%) and in a relationship (50.53%), as presented in Table 1. Insufficient level of exercise (PA < 150 min per last week) was demonstrated most frequently among university students (*n* = 2134, 66.07%) compared to a sufficient level of PA (*n* = 1096, 33.93%). Exposure to the coronavirus during the second wave of the COVID-19 pandemic was as follows: 30.90% of participants had symptoms of coronavirus infection (Exposure 1), 14.68% of them were tested for coronavirus (Exposure 2), 1.89% were hospitalized because of the COVID-19 (Exposure 3), and 12.91% were in strict quarantine for at least 14 days (Exposure 4). A total of 50.96% of students had family members or friends who were infected (Exposure 5), 7.83% of individuals had experienced the death of a loved one or a relative (Exposure 6), 24.33% of participants had experienced a job loss during the pandemic (Exposure 7), and 66.50% of the study sample reported worsening economic status (Exposure 8). A comparison of Polish and Ukrainian participants is presented in Table 1.

### 2.4. Statistical Analysis

Categorical variables, such as gender, relationship status, physical activity, and exposure to the COVID-19 pandemic, were compared between Polish and Ukrainian university students’ samples using contingency tables and Pearson’s χ^2^ test of independence, with φ or Cramer’s V to assess effect size (depending on several categories). The preliminary descriptive statistics were examined for continuous variables, including mean (*M*), standard deviation (*SD*), median (*Mdn*.), skewness, and kurtosis. Since the sample size is large, and skewness and kurtosis do not exceed the range +1 and −1 in physical and mental health variables (i.e., life satisfaction, physical health, stress, coronavirus-related PTSD, anxiety, depression, religiosity, and perceived positive effects of lockdown), the parametric properties are sufficient for using parametric statistical analyses.

The independent samples Student’s *t*-test was conducted to examine differences between gender groups (men and nonbinary, women), countries (Poland, Ukraine), and PA levels (sufficient, insufficient) in indicators of well-being, such as life satisfaction, physical health, stress, coronavirus-related PTSD, anxiety, depression, religiosity, and perceived positive effects of lockdown. The effect size was assessed by Cohen’s *d* statistic. However, if variances were not equal between samples (assessed by Levene’s *F* test of variance homogeneity), the Welsh *t*-test was performed as an equivalent to Student’s *t*-test. Association between continuous variables was assessed using Pearson’s correlations. Furthermore, the multiple linear regression (enter method) was performed for life satisfaction as an explained variable, and physical health, stress, coronavirus-related PTSD, anxiety, depression, religiosity, and perceived positive effects of lockdown as predictors. In addition, demographic variables were added to the regression model as confounders, including age (as a continuous variable), gender (men and nonbinary = 0, women = 1), relationship status (0 = in relationship, 1 = single), country (Poland = 0, Ukraine = 1), and PA (insufficient = 0, sufficient = 1).

Finally, the path model was examined using structural equation modeling (SEM) to find various paths leading to the subjective well-being of university students during the second wave of the COVID-19 pandemic. The maximum likelihood (ML) estimation method was implemented in the sample of 3230 participants. The model included stress, age, religiosity, and perceived positive effects of lockdown as exogenous covariates; gender, relationship status, country, and PA as exogenous factors; and life satisfaction, physical health, anxiety, depression, and coronavirus-related PTSD as endogenous variables. An adjusted bias-corrected bootstrapping technique with 1000 replications was performed to increase the accuracy of estimates and parameters for direct and indirect effects.

All structural models were evaluated using several goodness-of-fit criteria [94], such as maximum likelihood (ML) χ^2^, *df* and *p*-value (the ratio χ^2^/*df* < 5 representing good fit), standardized root mean squared residual (SRMR < 0.08 is acceptable), root mean square error of approximation (adequate fit if RMSEA ≤ 0.08), and comparative fit index (CFI ≥ 0.90 meaning adequate fit). The configural measurement invariance was examined using multigroup structural equation modeling (MGSEM) to check whether the path model varies across countries (Poland and Ukraine). For an adequate sample size (*n* > 300), Chen [95] suggests a change of >−0.010 in CFI, supplemented by a change of >0.015 in RMSEA or a change of >0.030 in SRMR, which suggests non-invariance. All statistical analyses were conducted using JAMOVI software ver. 2.2.5 [96,97,98,99].

## 3. Results

### 3.1. Prevalence of Physical and Mental Health Indicators among Polish and Ukrainian University Students during the Second Wave of the COVID-19 Pandemic

The frequency of occurrence in particular categories of life satisfaction, physical health, perceived stress, coronavirus-related PTSD, anxiety, and depression were compared between Polish and Ukrainian samples of university students (Table 2). The Pearson’s χ^2^ test of independence showed significant country differences in all variables. More Polish university students were dissatisfied with their lives, and fewer were satisfied than Ukrainian students. Many more Ukrainian university students self-rated their health as excellent than Polish students did. Extremely low stress was found more frequently among Ukrainian individuals, while extremely high stress was more often reported in the Polish sample. The risk of coronavirus-related PTSD (cut-off 44) was less frequent in the Ukrainian than in the Polish group. No anxiety risk was reported more frequently among Ukrainian university students, while severe anxiety risk was more often in the Polish sample. Similarly, no depression risk or mild depression was presented more frequently in Ukrainian university students. In contrast, moderate, moderately severe, and severe risk of depression was reported less often than in the Polish sample of university students.

### 3.2. Differences between Polish and Ukrainian University Students in Physical and Mental Health

Differences between Polish and Ukrainian university students were assessed using the independent samples Student’s and Welsh’s *t*-test. The results are presented in Table 2. The Polish sample showed significantly worst self-reported physical health, higher levels of stress, coronavirus-related PTSD, anxiety, depression, and religiosity than the Ukrainian sample, but the effect size was small (Cohen’s *d* ranged between 0.12 to 0.39). Consequently, Ukrainian students demonstrated higher life satisfaction and positive effects of lockdown than Polish students, with small and medium effect sizes, respectively (Table 3).

### 3.3. Gender Differences in Physical and Mental Health

Gender differences were examined using the independent samples Student’s and Welsh’s *t*-test, respectively (Table 4). Women showed significantly higher scores than men in all indices of mental and physical health. The results indicate that women present worse physical health than men and also higher levels of stress, coronavirus-related PTSD, anxiety, and depression. In contrast, female university students significantly better perceived the positive effects of the COVID-19 pandemic and reported higher life satisfaction and religiosity compared to males. However, the effect size for all differences is small (Cohen’s *d* ranges between −0.11 to −0.34).

### 3.4. Differences in Physical and Mental Health between Physically Active and Inactive University Students

Physical activity of university students representing sufficient and insufficient levels (cut-off PA = 150 min per week) was compared regarding the indices of physical and mental health using the independent samples Student’s and Welsh’s *t*-test, respectively. As presented in Table 5, physically active participants have significantly better self-rated physical health and subjective well-being level, perceive more positive effects of lockdown, have lower levels of stress, and have fewer symptoms of PTSD related to the COVID-19 pandemic, anxiety, and depression than people with insufficient PA. However, the effect size was small for all these differences (Cohen’s *d* ranged between 0.12 to 0.32). No significant differences in religiosity were found between people of various levels of physical activity.

### 3.5. Associations between Subjective Well-Being and Physical and Mental Health

**Figure 1:**
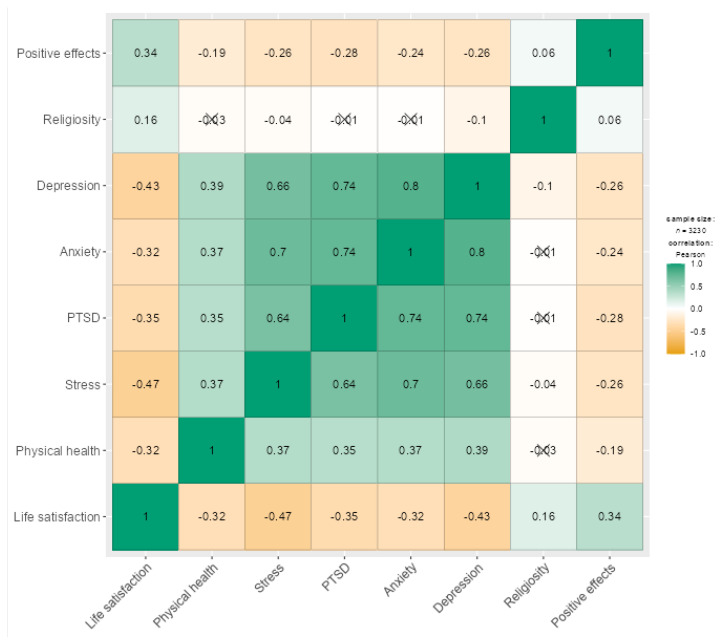
Correlation matrix (heatmap) with Pearson’s *r* coefficients (*p* < 0.05, *N* = 3230). Note. PTSD = post-traumatic stress disorder related to the COVID-19 pandemic. Deleted coefficients are not significant (*p* > 0.05). Positive correlations (ranging between 0 and +1) are in green, while negative associations (ranging between 0 and −1) are in orange.

Association between the physical and mental health indicators was examined using Pearson’s correlation (Figure 1). Life satisfaction is positively related to religiosity and perceived positive effects of lockdown, while it is negatively associated with physical health, stress, coronavirus-related PTSD, anxiety, and depression (*p* < 0.001). Religiosity is not related to physical health, anxiety, depression, and PTSD, while it is weakly positively related to stress (*p* < 0.05) and positive effects of the pandemic (*p* < 0.01). A high level of perceived positive pandemic effects was also associated with better physical health and fewer symptoms of stress, coronavirus-related PTSD, anxiety, and depression (*p* < 0.001). Physical health, stress, coronavirus-related PTSD, anxiety, and depression were positively correlated with each other at *p*-values less than 0.001.

The multiple linear regression was conducted to find predictors of life satisfaction among continuous (age, physical health, stress, coronavirus-related PTSD, anxiety, depression, religiosity, and positive effects of lockdown) and categorical variables (gender, country, relationship status, PA). The assumptions of regression were acceptable, including multicollinearity assessed by tolerance (<0.1) and variance inflation factor (VIF < 4), autocorrelation (0.03, Durbin-Watson *d* = 1.95, *p* = 0.126), no influential cases biasing the model of regression (Cook’s distance < 1), heteroskedasticity (Goldfield-Quandt = 1.01, *p* = 0.385), and multivariate normality (Anderson-Darling = 0.48, *p* = 0.233). The results of the regression are shown in Table 6. All variables are significant predictors of life satisfaction except coronavirus-related PTSD. Model can explain 36% of life satisfaction variance, *R* = 0.60, *R*^2^ = 0.36, *F*(12, 3217) = 154, *p* < 0.001.

### 3.6. Path Model for Predictors of Life Satisfaction among University Students during the Second Wave of the COVID-19 Pandemic

**Figure 2:**
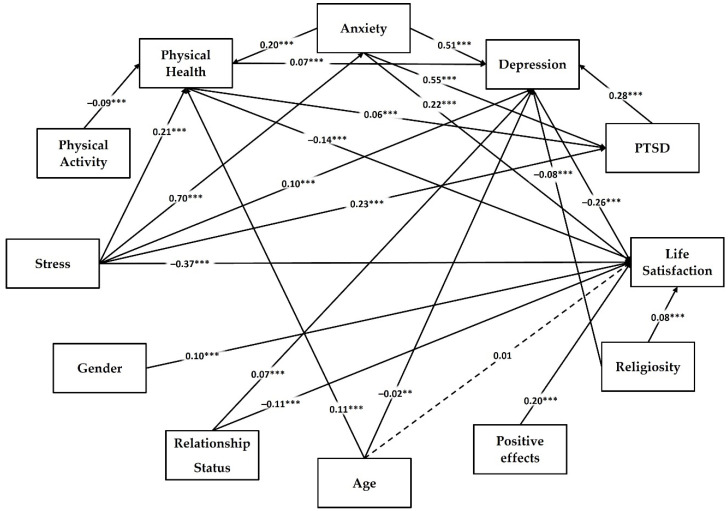
Path model with standardized regression weights for predictors of well-being among university students during the second wave of the COVID-19 pandemic (*n* = 3230); ** *p* < 0.01; *** *p* < 0.001.

The path model was explored based on the previous literature and on the current associations found in the study sample using structural equation modeling (SEM). The path model showed the following goodness of fit indices: χ^2^(20) = 148, *p* < 0.001, χ^2^/*df* = 7.4, RMSEA = 0.045 (0.038, 0.051), SRMR = 0.023, CFI = 0.989, TLI = 0.974, and GFI = 0.999. Regression coefficients are presented in Table 7, Figure 2, and Table S1 (in Supplementary Materials). Life satisfaction is predicted by better physical health (β = −0.14, *p* < 0.001), low stress (β = −0.37, *p* < 0.001), high anxiety (β = 0.22, *p* < 0.001), low depression (β = −0.26, *p* < 0.001), high scores in religiosity (β = 0.08, *p* < 0.001), and perceived positive effects of lockdown (β = 0.20, *p* < 0.001). In addition, some demographic variables, such as female gender (β = 0.10, *p* < 0.001) and being in a relationship (β = −0.11, *p* < 0.001), were associated with life satisfaction. Age was not a significant predictor of life satisfaction in the path model. All predictors explained 35% of life satisfaction variance, *R*^2^ = 0.35, Wald’s χ^2^(9) = 1896, *p* < 0.001. Physical health was predicted by stress (β = 0.21, *p* < 0.001), PA (β = −0.09, *p* < 0.001), older age (β = 0.11, *p* < 0.001), and anxiety (β = 0.20, *p* < 0.001); *R*^2^ = 0.18, Wald’s χ^2^(4) = 814, *p* < 0.001. Coronavirus-related PTSD was predicted by poor physical health (β = 0.06, *p* < 0.001), high stress (β = 0.23, *p* < 0.001), and high anxiety (β = 0.55, *p* < 0.001); *R*^2^ = 0.58, Wald’s χ^2^(3) = 3564, and *p* < 0.001. Anxiety was predicted by stress (β = 0.70, *p* < 0.001); *R*^2^ = 0.49, Wald’s χ^2^(1) = 3008, and *p* < 0.001. Significant predictors of depression were poor physical health (β = 0.07, *p* < 0.001), high stress (β = 0.10, *p* < 0.001), high anxiety (β = 0.51, *p* < 0.001), severe symptoms of coronavirus-related PTSD (β = 0.28, *p* < 0.001), low self-rated religiosity (β = −0.08, *p* < 0.001), younger age (β = −0.02, *p* < 0.01), and single relationship status (β = 0.07, *p* < 0.001); *R*^2^ = 0.72, Wald’s χ^2^(7) = 8282, and *p* < 0.001. All indirect effects were significant, as shown in Table 8.

### 3.7. Multigroup Analysis of Country Invariance for Path Model

The multigroup structural equation modeling (MGSEM) was conducted for the path model, examining measurement invariance across Polish and Ukrainian groups of university students. The configural invariance was tested to check whether cross-cultural differences exist in the regression loadings, predictive paths, and correlations of the model determining life satisfaction. MGSEM demonstrates a better absolute fit for the model constrained equally across Polish and Ukrainian samples than the baseline unconstrained model for the total sample if considering χ^2^(64) = 298, *p* < 0.001; χ^2^/*df* = 4.65. However, slightly but not significant worse fit indices were found in the constrained model (compared to unconstrained) for RMSEA = 0.048 (0.042, 0.053), SRMR = 0.035, GFI = 0.998, CFI = 0.979, and TLI = 0.970. The results indicate that the path model is invariant across countries [95]. All standardized regression weights were similar for both Polish and Ukrainian samples, as presented in Table 6, Tables S2 and S3, respectively, in the Supplementary Materials. In addition, all direct and indirect effects were found as significant (including the association between life satisfaction and age) in both country groups (see Tables S4 and S5 in the Supplementary Materials).

## 4. Discussion

### 4.1. Prevalence of Physical and Mental Health Problems

Consistent with hypothesis H1 and previous studies [6,19,20,21,22,23,24,25], we found a high prevalence of stress (PSS-10 ≥ 24, *n* = 1136, 35%), coronavirus-related PTSD (PCL-S ≥ 44, *n* = 741, 23%), anxiety (GAD-7 ≥ 10, *n* = 881, 27%), depression (PHQ-9 ≥ 10, *n* = 1240, 48%), low life satisfaction (SWLS ≤ 20, *n* = 1290, 40%), and fair or poor physical health (*n* = 227, 7%) in the total sample of university students (*n* = 3230). The results of this study are consistent with previous studies to some extent. Our previous international study reported the prevalence of perceived stress, anxiety, and depression symptoms at 61%, 30%, and 40%, respectively, among university students from nine countries (including Ukraine and Poland) during the first phase of the pandemic (May–July 2020) [5]. Therefore, depression and anxiety did not change significantly, while stress decreased in the second wave of the pandemic compared to the first wave. In addition, the prevalence of coronavirus-related PTSD (PCL-S ≥ 44) was much higher (32.70%) in the international sample of university students from six countries than in the current group [46]. The global prevalence of stress symptoms was reported in 37%, depression in 28%, anxiety in 27%, post-traumatic stress in 24%, and somatic symptoms in 31%, while low well-being was evidenced in 29% of adults from 32 countries worldwide, as it was shown in the systematic review and meta-analysis [23]. Although stress, PTSD, and anxiety are similar between this study and the previous study [23], the university student sample in the current study reported low life satisfaction levels more frequently and moderate-to-severe depression risk, while poor physical health was reported less frequently. Another review and meta-analysis found depressive symptoms among 34% of university students, while anxiety risk was reported in 32% of them [3]. In contrast to the study by Deng et al. [3], the present sample demonstrated significantly higher depression symptoms.

Significant changes between the first and third waves of the COVID-19 pandemic were previously reported among Polish university students [6]. The frequency of individuals with moderate anxiety risk was 38.4% during the first wave of the pandemic, while it rose to 46.3% during the third. In addition, more individuals were dissatisfied with their life during the third (49.54%) than the first (37.44%) stage of the pandemic. Similarly, the frequency of students with somatic symptoms increased significantly from the first (6.5%) to the third (16.9%) wave of the COVID-19 pandemic [6]. However, the prevalence of perceived stress decreased from the first (80.7%) to the third (55.2%) wave of the pandemic among Polish university students [6], which seems to confirm the trends found in the current study.

We found significant differences between Polish and Ukrainian samples in all dimensions of physical and mental health, including perceived stress, coronavirus-related PTSD, anxiety and depression symptoms, physical health, and satisfaction with life levels, consistent with hypothesis H1. In particular, a high prevalence of stress was more frequent in the Polish sample (*n* = 656, 41%) than in the Ukrainian sample (*n* = 480, 29%). Similarly, a risk of coronavirus-related PTSD (PCL-S ≥ 44) was reported in 28% of Polish students (*n* = 447) and 18% of their Ukrainian counterparts (*n* = 294). Moderate-to-severe anxiety symptoms were found among 488 (31%) Polish students and 393 (24%) Ukrainian. In addition, a moderate-to-severe depression risk was shown in 43% of Polish students (*n* = 685), while it was shown in 34% of Ukrainian individuals (*n* = 555). Furthermore, fair or poor physical health was demonstrated in 9% of Polish participants (*n* = 144) and only 5% of Ukrainian (*n* = 81). Compared to our previous research [32,63], the level of anxiety and depression did not change significantly between the first and second waves of the pandemic among Ukrainian university students. In contrast, stress and anxiety decreased, but somatic symptoms increased successively in the Polish sample. In the sample of Polish university students, moderate-to-severe anxiety symptoms were found in 35% and high perceived stress in 56%, while 6% self-rated physical health as poor during the early pandemic stage [32]. Furthermore, moderate anxiety symptoms were found among 24% of Ukrainian university students, while moderate depression was found among 32% of them during the first wave of the COVID-19 pandemic [63].

We have found relatively high prevalence rates of people dissatisfied with their lives (SWLS scores ≤ 20) among Polish university students (*n* = 745, 47%) and a significantly lower number in Ukrainian students (*n* = 545, 33%). The prevalence of life dissatisfaction during the first pandemic wave was 37% in the Polish sample and 40% in the Ukrainian group of students as suggested in our previous study [47]. So, we can conclude that the frequency of dissatisfied students significantly increased from the first to the second wave of the pandemic in the Polish sample while it slightly decreased in the Ukrainian group.

### 4.2. Country, Gender, and Exercise Differences in Mean Levels of Mental and Physical Health and Well-Being

We performed Student’s *t*-tests several times to examine intergroup differences in mental and physical health and well-being among university students regarding country, gender, and PA levels. Consistent with hypothesis H2 and previous studies [2,3,4,5,23,30,31,45,46,47], country differences were found in the current research. Confirming the analysis of the prevalence of mental and physical problems presented above, the study showed that Polish university students reported the worst physical health and life satisfaction, with higher levels of stress, coronavirus-related PTSD, anxiety, depression, and religiosity, compared to the Ukrainian sample. In addition, fewer positive effects of the pandemic were perceived in Polish university students than among Ukrainian participants. The generally better sense of well-being of Ukrainian students is consistent with previous research [47,100]. According to the Gallup World Poll survey results, Poland ranked position 39, while Ukraine ranked 69 in perceived sense of happiness among 149 countries during COVID-19 [100].

Furthermore, university students from Poland showed significantly lower life satisfaction than their counterparts from Ukraine (*p* < 0.001) during the first wave of the COVID-19 pandemic [47]. Considering the other mental health dimensions, Ukrainian university students may demonstrate higher resiliency than their counterparts from Poland. The second explanation is that the Ukrainians assess the current situation positively compared to the previous decades when the political and socio-economic status depended on Russia or even earlier on the communist system of the totalitarian Soviet Union. University students from Ukraine may currently have many more possibilities for development and self-realization than their parents and grandparents. Ukraine has undergone many systemic reforms that give hope for a better tomorrow. So, maybe Ukrainian students are more optimistic about the future than Polish university students. In addition, Ukrainians may believe in their coping skills and self-efficacy to a greater extent than Polish students. More research is necessary in the future to explain the discrepancy in well-being between these two countries.

Women demonstrate worse mental and physical health than men in the study, including such dimensions as higher stress, coronavirus-related PTSD, anxiety, depression, and somatic symptoms. The results of this study are consistent with Hypothesis H3 and previous research [2,3,5,6,26,27,28,29,30,31,32,33,34,35,36]. Women are included in a high-risk group for most mental health problems, being more sensitive to environmental changes, using more negative emotions, and coping with stressful situations more emotionally than men. Moreover, the study showed that women reported better life satisfaction and religiosity than men and perceived more positive effects of the pandemic. The results indicate that women are more optimistic, perceive more possibilities to be happy, and have higher resilience than men. These properties can help women cope with difficulties and support high resistance in stressful and demanding living conditions.

According to previous results [47,53,54,55,56,59,60,63,65,66,101,102], we assumed in hypothesis H4 that university students with insufficient levels of exercise (PA ≤ 150 min per week) demonstrate poorer physical and mental health compared to their counterparts with a sufficient PA level. In general, most university students represented an insufficient level of PA (66%). Hypothesis H4 was confirmed since we found worse physical health and life satisfaction, while higher stress, coronavirus-related PTSD, anxiety, and depression symptoms among participants with insufficient PA levels than among those exercising systematically. It was found in previous studies that regular and sufficient levels of PA are beneficial for physical health by improving sleep quality, cardiovascular, respiratory, and immune systems, enhancing aerobic capacity and endurance, and reducing cholesterol and body weight [103,104,105]. Therefore, exercises are prescribed for patients with various diseases [106]. It was also evidenced during the COVID-19 pandemic that high PA levels improved sleep quality and mitigated the mental health burden related to a lockdown, such as increased stress, negative emotions, anxiety, depression, or worsening sense of well-being [47,53,54,55,56,59,61,63,65,66,101,102]. Physical activity can improve the function of immune and respiratory systems and may predict coronavirus infection [104,107].

Furthermore, Sochacka and Zdziarski [56] found a strong correlation between PA and the well-being of university students from Poland. Finally, Wang et al. [108] found that sleep quality mediates the relationship between PA and health-related quality of life among Chinese adults during the COVID-19 pandemic. Therefore, sleep quality could be included in further studies. The present study is in line with a vast body of literature that PA may mitigate the negative consequences of the pandemic and should be considered an essential part of activities during the lockdown.

### 4.3. Associations between the Subjective Well-Being and Physical and Mental Health during the Second Wave of the COVID-19 Pandemic

The results of this study showed for the first time, to the best of our knowledge, that various paths can contribute to life satisfaction of university students during the COVID-19 pandemic. In contrast to previous studies that examined associations between selected aspects of health, we have found a complex model to explain the interplay between physical and mental health dimensions in a biopsychosocial model of health and well-being [74,75,76]. Due to the holistic model of health and well-being, all biological, psychological, and social factors mutually affect each other. To fully understand the mechanism of a disease or health status, various aspects should be included in the model. The present path model examined the interactions between factors such as age, biological sex, and global exposure to the COVID-19 pandemic (the study was performed during the second wave). Psychological and behavioral factors consisted of perceived health status, stress, PTSD, anxiety, depression, religiosity, physical activity, and perceived positive effects of the pandemic. The socio-cultural factors studied included gender, relationship status, and country.

Apart from the coronavirus-related PTSD, all variables assumed in hypothesis H5 were confirmed in the study as significant predictors of life satisfaction, considering the multivariate linear regression model. The results of this study are consistent with previous studies that found numerous associations between these variables [2,3,5,6,25,26,27,28,29,30,31,32,33,34,35,36,47,49,50,51,52,67,68,69,70,71,72,73]. For example, longitudinal research confirmed that in the three waves of the COVID-19 pandemic high anxiety risk was related negatively to life satisfaction and positively related to high levels of stress among Polish university students [6]. In general, a high level of adverse somatic and mental symptoms (e.g., low self-rated physical health, high stress, anxiety, and depression) decreased life satisfaction both before [70,71,72] and during the pandemic [6]. Perceived worst health status was also related to high stress, anxiety, and low life satisfaction among Polish university students during the first wave of the pandemic [32]. In addition, people with chronic diseases and severe symptoms of COVID-19 showed a lower life satisfaction level than healthy individuals [36]. A recent study showed that poor self-rated health status was a predictor of low satisfaction in life in a large international sample of university students from nine countries [47]. Meanwhile, the study found that coronavirus-related PTSD was not related to life satisfaction, which may be a consequence of the very low prevalence of PTSD in the university student sample. As shown in Table 1, only a few participants were directly exposed to the coronavirus infection. In the study sample, 31% reported having symptoms of coronavirus infection. Still, only 15% were tested for COVID-19, 2% were hospitalized because of coronavirus infection, and 13% had to be quarantined for at least 14 days. Even though someone in the student sample tested positive for COVID-19, it was likely not a big problem since most students were young, and 93% of them self-rated their physical health positively.

On the other hand, among the hidden positive effects of the COVID-19 pandemic, many people’s social and cultural lives improved during the lockdown. For example, an increase in communication, social integration, and social cohesion was observed in families coping with hardship during the pandemic [76]. People reported spending more time with family and loved ones and had become more invested in the relationship, increasing sexual behavior and relationship happiness [17]. Many people also found time to develop their interests and learn new skills, provide a more active and healthier lifestyle, and develop interests, art activities, and hobbies [15]. In addition, a significant increase in technological innovation, creativity, and handicraft production was found during the lockdown as well as reduced unhealthy and harmful behavior (such as substance use, gambling, commercial sex, violence, and suicide). In particular, more positive aspects of the COVID-19 pandemic were evidenced in socially advantaged individuals, while more negative effects were observed in socially disadvantaged people [18].

The culmination of statistical analysis in the study was the path model. We found several direct predictors of satisfaction with life, such as stress, anxiety, depression, physical health, perceived positive effects of the pandemic, religiosity, gender, and relationship status. Interestingly, coronavirus-related PTSD is not directly associated with life satisfaction, but this relationship is mediated by depression. Older age and high PA level are related to a better appraisal of life satisfaction indirectly by self-reported health status. Low religiosity, younger age, and single relationship status can negatively affect life satisfaction via depression. Stress links to life satisfaction directly and via various paths through physical health, anxiety, depression, and coronavirus-related PTSD. Physical fitness can change the level of life satisfaction directly and indirectly affect coronavirus-related PTSD and depression. Anxiety contributes to life satisfaction directly and also through physical health, depression, and coronavirus-related PTSD. Depression is directly related to life satisfaction and acts in the model as the most frequent mediator of various associations.

Furthermore, these associations seem independent of the country, as evidenced by multigroup measurement invariance in the path model and contradict hypothesis H5. Although numerous country differences were found in life satisfaction and all physical and mental health dimensions, these discrepancies do not contribute to the pattern of associations between these variables. In both Polish and Ukrainian samples of university students, similar regression weights and the same mediation effects were confirmed. The result of this study may indicate that the path model is universal. It means that all people, regardless of culture, language, or political climate, link the same mental and physical health variables in the same way and with similar strengths. However, more research is necessary to verify this speculation.

### 4.4. Limitation of the Study

Considering some limitations of this study, the cross-sectional design may be seen as a source of bias in the regression analysis and path model. Longitudinal studies are necessary to fully confirm causal associations between variables. Although the sample size was large in both Ukrainian and Polish groups of university students, a convenience sampling method may be a source of bias. In addition, a web-based survey may be a limitation of this study. Future studies should be performed in a more representative sample and by using other methods of recruiting participants and other forms of questionnaires (e.g., paper-and-pencil, telephone-based survey).

## 5. Conclusions

The study confirmed to a large extent all assumed hypotheses. There was a high prevalence of stress, coronavirus-related PTSD, anxiety, depression, and a low level of life satisfaction and physical health among university students during the second wave of the COVID-19 pandemic. Moreover, Polish university students presented worse well-being and physical and mental health than their Ukrainian counterparts. Not only do countries differ in the mean level of physical and mental health dimensions among university students, but also women and people with insufficient levels of exercise are at higher risk of adverse consequences of the COVID-19 pandemic. These groups should be a target population for prevention programs during global crises such as the pandemic. The study evidenced that a high level of life satisfaction can be directly predicted by low perceived stress, anxiety, and depression and better physical health, sufficient PA levels, high religiosity, and more perceived positive effects of the pandemic. In addition, numerous mediating effects were found, which showed that subjective well-being may be affected by various paths leading through physical health, anxiety, and depression in particular. The pattern of associations found in the study seems universal as it works similarly for the Ukrainian and Polish participants, regardless of intercultural differences.

The greatest contribution to the level of life satisfaction includes the following variables: (1) stress, (2) depression, (3) anxiety, (4) perceived positive effects of the COVID-19 pandemic, (5) general self-rated physical health, (6) relationship status, (7) gender, (8) religiosity, and (9) age. Therefore, prevention programs on campuses should be primarily aimed at lowering levels of stress, depression, and anxiety. It is recommended to introduce to the curriculum of all faculties of study classes that will prevent mental and physical health problems, with a wide range of classes presenting strategies for coping with stress, controlling negative emotions and anxiety, promoting a healthy lifestyle with special attention on easy access to physical activity on campuses (e.g., special infrastructure, more gyms and a rich offering of various forms of recreation and sports, and relaxation techniques). Since perceived positive effects of the pandemic have a significant effect on life satisfaction, some techniques promoting positive and optimistic thinking, affirmation, and transforming negative thinking and feelings into positive ones should be helpful as well. The COVID-19 pandemic has shown that schools, universities, and workplaces should be more responsible for public health. Systemic change is needed to improve the physical and mental health of populations around the world based on the biopsychosocial model of well-being.

## Figures and Tables

**Table 1:** A comparison of Polish and Ukrainian university students in gender, relationship status, physical activity, and exposure to the COVID-19 pandemic.

	Sample				Total Sample		χ^2^	*df*	*p*	φ
	Polish		Ukrainian							
Variable	*n*	%	*n*	%	*n*	%				
**Gender**							78.98	2	<0.001	0.156 _V_
Men	748	23.16	528	16.35	1276	39.51				
Women	815	25.23	1097	33.96	1912	59.20				
Nonbinary	18	0.56	24	0.74	42	1.30				
**Relationship status**							49.74	1	<0.001	0.124
In a couple	899	27.833	733	22.69	1632	50.53				
Single	682	21.12	916	28.36	1598	49.47				
**Physical activity**							4.82	1	0.028	−0.039
Insufficient	1015	31.42	1119	34.64	2134	66.07				
Sufficient	566	17.52	530	16.41	1096	33.93				
**Exposure 1**							81.48	1	<0.001	0.159
No	1211	37.49	1021	31.61	2232	69.10				
Yes	370	11.46	628	19.44	998	30.90				
**Exposure 2**							15.06	1	<0.001	0.068
No	1388	42.97	1368	42.35	2756	85.33				
Yes	193	5.98	281	8.70	474	14.68				
**Exposure 3**							2.52	1	0.112	−0.028
No	1545	47.83	1624	50.28	3169	98.11				
Yes	36	1.12	25	0.77	61	1.89				
**Exposure 4**							18.62	1	<0.001	0.076
No	1418	43.90	1395	43.19	2813	87.09				
Yes	163	5.05	254	7.86	417	12.91				
**Exposure 5**							13.06	1	<0.001	−0.064
No	724	22.42	860	26.63	1584	49.04				
Yes	857	26.53	789	24.43	1646	50.96				
**Exposure 6**							0.17	1	0.679	−0.007
No	1454	45.02	1523	47.15	2977	92.17				
Yes	127	3.93	126	3.90	253	7.83				
**Exposure 7**							4.65	1	0.031	−0.038
No	1170	36.22	1274	39.44	2444	75.67				
Yes	411	12.72	375	11.61	786	24.33				
**Exposure 8**							2.09	1	0.148	0.025
No	549	17.00	533	16.50	1082	33.50				
Yes	1032	32.00	1116	34.55	2148	66.50				

Note: Exposure = exposure to COVID-19 to assess the consequences of COVID-19: Exposure 1 = symptoms of coronavirus infection; Exposure 2 = testing for coronavirus; Exposure 3 = hospitalized; Exposure 4 = being in strict quarantine for at least 14 days; Exposure 5 = family or friend infected; Exposure 6 = death of a loved one or a relative; Exposure 7 = job loss; Exposure 8 = worsening economic status; _V_ = Cramer’s V as the effect size for more than two categories of gender.

**Table 2:** Frequencies of particular categories of life satisfaction, physical health, perceived stress, coronavirus-related PTSD, anxiety, and depression (*N* = 3230).

Variable	Sample				Total Sample (*n* = 3230)		χ^2^	*df*	*p*	φ
	Polish (*n* = 1581)		Ukrainian (*n* = 1649)							
	*n*	%	*n*	%	n	%				
**Life satisfaction**							110.55	6	<0.001	0.19
Extremely dissatisfied	54	1.67	26	0.81	80	2.48				
Dissatisfied	230	7.12	120	3.72	350	10.84				
Slightly dissatisfied	384	11.89	321	9.94	705	21.83				
Neutral	77	2.38	78	2.42	155	4.80				
Slightly satisfied	485	15.02	515	15.94	1000	30.96				
Satisfied	280	8.67	455	14.09	735	22.76				
Extremely satisfied	71	2.20	134	4.15	205	6.35				
**Physical health**							217.91	4	<0.001	0.26
Excellent	144	4.46	454	14.06	598	18.51				
Very good	641	19.85	431	13.34	1072	33.19				
Good	652	20.19	681	21.08	1333	41.27				
Fair	130	4.03	77	2.38	207	6.41				
Poor	14	0.43	6	0.19	20	0.62				
**Perceived stress**							71.61	4	<0.001	0.15
Extremely low	158	4.89	232	7.18	390	12.07				
Low	300	9.29	403	12.48	703	21.77				
Average	467	14.46	534	16.53	1001	30.99				
High	351	10.87	307	9.51	658	20.37				
Extremely high	305	9.44	173	5.36	478	14.80				
**Coronavirus-related PTSD**							49.80	1	<0.001	−0.12
No	1134	35.11	1355	41.95	2489	77.06				
Yes	447	13.84	294	9.10	741	22.94				
**General anxiety disorder**							36.52	3	<0.001	0.11
No anxiety	595	18.42	711	22.01	1306	40.43				
Mild anxiety	498	15.42	545	16.87	1043	32.29				
Moderate anxiety	280	8.67	276	8.55	556	17.21				
Severe anxiety	208	6.44	117	3.62	325	10.06				
**Major depression**							36.85	4	<0.001	0.11
No depression	440	13.62	569	17.62	1009	31.24				
Mild depression	456	14.12	525	16.25	981	30.37				
Moderate depression	351	10.87	309	9.57	660	20.43				
Moderately severe	214	6.63	167	5.17	381	11.80				
Severe depression	120	3.72	79	2.45	199	6.16				

**Table 3:** A comparison of Ukrainian and Polish samples in mental and physical health.

Variable	Polish (*n* = 1581)		Ukrainian (*n* = 1649)		*t*	*df*	*p*	Δ*M*	*d*
	*M*	*SD*	*M*	*SD*					
Life satisfaction	20.55	6.08	22.86	5.82	−11.01 _a_	3205	<0.001	−2.31	−0.39
Poor physical health	2.51	0.81	2.24	0.92	8.88 _a_	3201	<0.001	0.27	0.31
Stress	21.56	7.53	19.46	7.16	8.10 _a_	3201	<0.001	2.10	0.29
Coronavirus-related PTSD	36.90	13.37	32.91	11.48	9.08 _a_	3112	<0.001	3.99	0.32
Anxiety	7.29	5.58	6.27	4.97	5.47 _a_	3151	<0.001	1.02	0.19
Depression	9.19	6.43	7.89	5.97	5.97 _a_	3185	<0.001	1.30	0.21
Positive lockdown effects	14.02	5.82	17.16	5.91	−15.20 _b_	3228	0.001	−3.14	−0.54
Religiosity	1.58	0.98	1.47	0.85	3.28 _a_	3123	0.001	0.11	0.12

Note. PTSD = post-traumatic stress disorder related to the COVID-19 pandemic; _a_ = Welsh’s *t*-test; _b_ = Student’s *t*-test.

**Table 4:** Gender differences in mental and physical health.

	Men (*n* = 1318)		Women (*n* = 1912)		*t*	*df*	*p*	Δ*M*	*d*
Variable	*M*	*SD*	*M*	*SD*					
Life satisfaction	21.18	6.09	22.11	6.01	−4.30 _b_	3228	<0.001	−0.93	−0.15
Poor physical health	2.30	0.88	2.43	0.87	−4.23 _b_	3228	<0.001	−0.13	−0.15
Stress	19.03	7.43	21.49	7.24	−9.38 _b_	3228	<0.001	−2.46	−0.34
Coronavirus-related PTSD	32.86	12.01	36.24	12.82	−7.64 _a_	2945	<0.001	−3.37	−0.27
Anxiety	5.83	5.20	7.43	5.27	−8.52 _b_	3228	<0.001	−1.60	−0.31
Depression	7.76	6.04	9.05	6.30	−5.80 _b_	3228	<0.001	−1.29	−0.21
Positive lockdown effects	15.21	6.06	15.90	6.06	−3.18 _b_	3228	0.002	−0.69	−0.11
Religiosity	1.36	0.99	1.64	0.85	−8.37 _a_	2556	<0.001	−0.28	−0.30

Note. PTSD = post-traumatic stress disorder related to the COVID-19 pandemic; _a_ = Welsh’s *t*-test; _b_ = Student’s *t*-test.

**Table 5:** A comparison of samples with sufficient and insufficient PA in mental and physical health.

	Insufficient PA (*n* = 2134)		Sufficient PA (*n* = 1096)		*t*	*df*	*p*	Δ*M*	*d*
Variable	*M*	*SD*	*M*	*SD*					
Life satisfaction	21.26	6.16	22.63	5.75	−6.25 _a_	2350	<0.001	−1.37	−0.23
Poor physical health	2.47	0.87	2.19	0.86	8.71 _b_	3228	<0.001	0.28	0.32
Stress	21.28	7.39	18.95	7.24	8.53 _b_	3228	<0.001	2.33	0.32
Coronavirus-related PTSD	35.80	12.67	33.04	12.27	5.91 _b_	3228	<0.001	2.75	0.22
Anxiety	7.26	5.35	5.82	5.07	7.73 _a_	2293	<0.001	1.75	0.29
Depression	9.12	6.26	7.37	6.00	7.63 _b_	3228	<0.001	1.75	0.28
Positive COVID-19 effects	15.37	5.99	16.11	6.19	−3.27 _b_	3228	0.001	−0.74	−0.12
Religiosity	1.52	0.92	1.54	0.93	−0.61 _b_	3228	0.541	−0.02	−0.02

Note. PTSD = post-traumatic stress disorder related to the COVID-19 pandemic; _a_ = Welsh’s *t*-test; _b_ = Student’s *t*-test.

**Table 6:** Multiple linear regression for life satisfaction.

Predictor	β	*B*	*SE*	95% *CI*		*t*	*p*
				Lower	Upper		
Age	0.05	0.08	0.03	0.03	0.14	2.88	0.004
Gender	0.20	1.20	0.19	0.83	1.57	6.34	<0.001
Relationship status	−0.22	−1.35	0.18	−1.70	−1.00	−7.59	<0.001
Country	0.19	1.14	0.20	0.75	1.53	5.72	<0.001
PA	0.10	0.58	0.19	0.21	0.94	3.09	0.002
Physical health	−0.13	−0.92	0.11	−1.13	−0.71	8.46	<0.001
Stress	−0.35	−0.29	0.02	−0.32	−0.25	−16.73	<0.001
PTSD	0.02	0.01	0.01	−0.01	0.03	0.69	0.489
Anxiety	0.21	0.23	0.03	0.17	0.29	7.60	<0.001
Depression	−0.26	−0.25	0.03	−0.30	−0.20	−9.61	<0.001
Positive effects	0.18	0.18	0.02	0.15	0.21	12.07	<0.001
Religiosity	0.08	0.55	0.10	0.36	0.73	5.70	<0.001

Note. PTSD = post-traumatic stress disorder related to the COVID-19 pandemic; *CI* = confidence interval.

**Table 7:** Standardized regression weights for path model in the total sample (*N* = 3230), Polish group (*n* = 1581) and Ukrainian group (*n* = 1649) of university students.

Variables		Total Sample β	Polish Sample β	Ukrainian Sample β
Dependent	Predictor			
Life satisfaction	Physical health	−0.14 ***	−0.13 ***	−0.15 ***
Life satisfaction	Anxiety	0.22 ***	0.22 ***	0.21 ***
Life satisfaction	Depression	−0.26***	−0.26 ***	−0.25 ***
Life satisfaction	Relationships	−0.11 ***	−0.11 ***	−0.11 ***
Life satisfaction	Religiosity	0.08 ***	0.09 ***	0.08 ***
Life satisfaction	Positive effects	0.20 ***	0.18 ***	0.19 ***
Life satisfaction	Gender	0.10 ***	0.09 ***	0.09 ***
Life satisfaction	Stress	−0.37 ***	−0.36 ***	−0.35 ***
Life satisfaction	Age	0.01	0.05 **	0.03 **
Physical health	PA	−0.09 ***	−0.11 ***	−0.09 ***
Physical health	Stress	0.21 ***	0.22 ***	0.19 ***
Physical health	Age	0.11 ***	0.09 ***	0.05 ***
Physical health	Anxiety	0.20 ***	0.22 ***	0.18 ***
PTSD	Physical health	0.06 ***	0.05 ***	0.06 ***
PTSD	Stress	0.23 ***	0.23 ***	0.22 ***
PTSD	Anxiety	0.55 ***	0.58 ***	0.55 ***
Anxiety	Stress	0.70 ***	0.69 ***	0.71 ***
Depression	Anxiety	0.51 ***	0.51 ***	0.50 ***
Depression	Religiosity	−0.08 ***	−0.09 ***	−0.08 ***
Depression	Stress	0.10 ***	0.10 ***	0.10 ***
Depression	Age	−0.02 **	−0.03 **	−0.02 **
Depression	Physical health	0.07 ***	0.07 ***	0.08 ***
Depression	Relationships	0.07 ***	0.07 ***	0.07 ***
Depression	PTSD	0.28 ***	0.27 ***	0.28 ***

Note. PTSD = post-traumatic stress disorder related to the COVID-19 pandemic; ** *p* < 0.01; *** *p* < 0.001.

**Table 8:** Estimation of path parameters for life satisfaction (*N* = 3230).

Path Parameter	*B*	*SE*	95% CI		β	*z*	*p*
			LL	UL			
PH ⇒ PTSD ⇒ Depression ⇒ LS	−0.03	0.01	−0.05	−0.02	0.00	−3.97	<0.001
PH ⇒ Depression ⇒ LS	−0.13	0.02	−0.18	−0.09	−0.02	−5.53	<0.001
GAD ⇒ PH ⇒ LS	−0.03	0.01	−0.04	−0.02	−0.03	−6.23	<0.001
GAD ⇒ PH ⇒ PTSD ⇒ Depression ⇒ LS	0.00	0.00	0.00	0.00	0.00	−3.68	<0.001
GAD ⇒ PH ⇒ Depression ⇒ LS	0.00	0.00	−0.01	0.00	0.00	−4.62	<0.001
GAD ⇒ PTSD ⇒ Depression ⇒ LS	−0.05	0.01	−0.06	−0.04	−0.04	−7.69	<0.001
GAD ⇒ Depression ⇒ LS	−0.15	0.02	−0.18	−0.11	−0.13	−8.55	<0.001
Relationships ⇒ Depression ⇒ LS	−0.22	0.04	−0.30	−0.15	−0.02	−5.68	<0.001
Religiosity ⇒ Depression ⇒ LS	0.14	0.02	0.10	0.19	0.02	6.41	<0.001
Stress ⇒ PH ⇒ LS	−0.03	0.00	−0.03	−0.02	−0.03	−6.41	<0.001
Stress ⇒ PH ⇒ PTSD ⇒ Depression ⇒ LS	0.00	0.00	0.00	0.00	0.00	−3.61	<0.001
Stress ⇒ PH ⇒ Depression ⇒ LS	0.00	0.00	−0.01	0.00	0.00	−4.76	<0.001
Stress ⇒ PTSD ⇒ Depression ⇒ LS	−0.01	0.00	−0.02	−0.01	−0.02	−7.39	<0.001
Stress ⇒ GAD ⇒ LS	0.13	0.02	0.10	0.16	0.16	7.82	<0.001
Stress ⇒ GAD ⇒ PH ⇒ LS	−0.02	0.00	−0.02	−0.01	−0.02	−6.22	<0.001
Stress ⇒ GAD ⇒ PH ⇒ PTSD ⇒ Depression ⇒ LS	0.00	0.00	0.00	0.00	0.00	−3.67	<0.001
Stress ⇒ GAD ⇒ PH ⇒ Depression ⇒ LS	0.00	0.00	0.00	0.00	0.00	−4.61	<0.001
Stress ⇒ GAD ⇒ PTSD ⇒ Depression ⇒ LS	−0.02	0.00	−0.03	−0.02	−0.03	−7.62	<0.001
Stress ⇒ GAD ⇒ Depression ⇒ LS	−0.08	0.01	−0.09	−0.06	−0.09	−8.42	<0.001
Stress ⇒ Depression ⇒ LS	−0.02	0.00	−0.03	−0.01	−0.03	−5.17	<0.001
Age ⇒ PH ⇒ LS	−0.03	0.01	−0.04	−0.02	−0.02	−5.51	<0.001
Age ⇒ PH ⇒ PTSD ⇒ Depression ⇒ LS	0.00	0.00	0.00	0.00	0.00	−3.33	<0.001
Age ⇒ PH ⇒ Depression ⇒ LS	0.00	0.00	−0.01	0.00	0.00	−4.35	<0.001
Age ⇒ Depression ⇒ LS	0.01	0.00	0.00	0.02	0.01	2.66	0.008
PA ⇒ PH ⇒ LS	0.17	0.04	0.10	0.24	0.01	4.72	<0.001
PA ⇒ PH ⇒ PTSD ⇒ Depression ⇒ LS	0.01	0.00	0.00	0.01	0.00	3.25	0.001
PA ⇒ PH ⇒ Depression ⇒ LS	0.02	0.01	0.01	0.04	0.00	3.91	<0.001
PTSD ⇒ Depression ⇒ LS	−0.04	0.00	−0.04	−0.03	−0.07	−8.29	<0.001

Note. LS = life satisfaction; PA = physical activity; PH = physical health; PTSD = post-traumatic stress disorder related to the COVID-19 pandemic; CI = confidence interval; LL = lower level; UL = upper level.

## Data Availability

The data are available to the corresponding author for a reasonable request.

## Supplementary Materials

The following supporting information can be downloaded at: https://www.mdpi.com/article/10.3390/jcm11164726/s1, Table S1: Parameters estimates for path model in the total sample (*N* = 3230); Table S2: Parameters estimates for path model in the Polish sample (*n* = 1581); Table S3: Parameters estimates for path model in the Ukrainian sample (*n* = 1649); Table S4: Estimation of path parameters for life satisfaction in Polish sample of university students (*n* = 1581); Table S5: Estimation of path parameters for life satisfaction in Ukrainian sample of university students (*n* = 1649).
