# Supplementary material for: A Path Model for Subjective Well-Being during the Second Wave of the COVID-19 Pandemic: A Comparative Study among Polish and Ukrainian University Students
Source: J Clin Med. 2022 Aug 12;11(16):4726. doi: 10.3390/jcm11164726 (PMC9410502; doi:10.3390/jcm11164726)
Supplement: Supplementary file 1 [file jcm-11-04726-s001.zip › jcm-1809959-supplementary.pdf]

Supplementary material

Table S1. Parameters estimates for path model in the total sample (*N* = 3230)

Variables		*B*	*SE*	95% CI		β	*z*	*p*
**Dependent**	**Predictor**			**Lower**	**Upper**			
Life satisfaction	Physical health	–0.98	0.11	–1.20	–0.76	–0.14	–8.77	< 0.001
Life satisfaction	Anxiety	0.25	0.03	0.19	0.31	0.22	7.95	< 0.001
Life satisfaction	Depression	–0.25	0.03	–0.30	–0.19	–0.26	–9.34	< 0.001
Life satisfaction	Relationships	–1.28	0.18	–1.63	–0.94	–0.11	–7.19	< 0.001
Life satisfaction	Religiosity	0.51	0.10	0.32	0.72	0.08	5.04	< 0.001
Life satisfaction	Positive effects	0.20	0.02	0.17	0.23	0.20	12.20	< 0.001
Life satisfaction	Gender	1.24	0.19	0.89	1.59	0.10	6.65	< 0.001
Life satisfaction	Stress	–0.30	0.02	–0.33	–0.27	–0.37	–18.03	< 0.001
Life satisfaction	Age	0.02	0.03	–0.03	0.07	0.01	0.69	0.488
Physical health	PA	–0.17	0.03	–0.23	–0.11	–0.09	–5.82	< 0.001
Physical health	Stress	0.03	0.00	0.02	0.03	0.21	9.71	< 0.001
Physical health	Age	0.03	0.00	0.02	0.04	0.11	7.08	< 0.001
Physical health	Anxiety	0.03	0.00	0.03	0.04	0.20	8.80	< 0.001
PTSD	Physical health	0.89	0.19	0.48	1.24	0.06	4.63	< 0.001
PTSD	Stress	0.39	0.03	0.34	0.45	0.23	13.87	< 0.001
PTSD	Anxiety	1.31	0.05	1.20	1.40	0.55	27.14	< 0.001
Anxiety	Stress	0.50	0.01	0.48	0.52	0.70	54.85	< 0.001
Depression	Anxiety	0.60	0.02	0.55	0.64	0.51	24.28	< 0.001
Depression	Religiosity	–0.57	0.07	–0.70	–0.44	–0.08	–8.72	< 0.001
Depression	Stress	0.08	0.01	0.06	0.10	0.10	6.32	< 0.001
Depression	Age	–0.04	0.01	–0.07	–0.01	–0.02	–2.82	0.005
Depression	Physical health	0.52	0.08	0.39	0.68	0.07	6.88	< 0.001
Depression	Relationships	0.87	0.12	0.64	1.11	0.07	7.20	< 0.001
Depression	PTSD	0.14	0.01	0.12	0.16	0.28	15.24	< 0.001

Note. PTSD = posttraumatic stress disorder related to the COVID-19 pandemic, *CI* = confidence interval.

Table S2. Parameters estimates for path model in the Polish sample (*n* = 1581)

Variables		*B*	*SE*	95% CI		β	*z*	*p*
**Dependent**	**Predictor**			**Lower**	**Upper**			
Life satisfaction	Physical health	–0.95	0.11	–1.14	–0.71	–0.13	–8.72	< 0.001
Life satisfaction	Anxiety	0.24	0.03	0.18	0.30	0.22	7.77	< 0.001
Life satisfaction	Depression	–0.24	0.03	–0.30	–0.20	–0.26	–9.37	< 0.001
Life satisfaction	Relationships	-1.35	0.18	–1.71	–1.01	–0.11	–7.63	< 0.001
Life satisfaction	Religiosity	–0.56	0.10	0.35	0.76	0.09	5.45	< 0.001
Life satisfaction	Positive effects	0.18	0.02	0.15	0.21	0.18	11.26	< 0.001
Life satisfaction	Gender	1.11	0.18	0.75	1.48	0.09	6.23	< 0.001
Life satisfaction	Stress	–0.29	0.02	–0.33	–0.26	–0.36	–17.19	< 0.001
Life satisfaction	Age	0.08	0.03	0.02	0.13	0.05	2.73	0.006
Physical health	PA	–0.18	0.03	–0.24	–0.12	–0.11	–5.98	< 0.001
Physical health	Stress	0.02	0.00	0.02	0.03	0.22	9.02	< 0.001
Physical health	Age	0.02	0.00	0.01	0.03	0.09	4.82	< 0.001
Physical health	Anxiety	0.03	0.00	0.03	0.04	0.22	8.71	< 0.001
PTSD	Physical health	0.76	0.19	0.39	1.15	0.05	3.93	< 0.001
PTSD	Stress	0.38	0.03	0.32	0.44	0.23	13.03	< 0.001
PTSD	Anxiety	1.33	0.05	1.22	1.41	0.58	27.67	< 0.001
Anxiety	Stress	0.50	0.01	0.48	0.52	0.69	56.90	< 0.001
Depression	Anxiety	0.60	0.02	0.55	0.64	0.51	25.51	< 0.001
Depression	Religiosity	–0.58	0.07	–0.71	–0.45	–0.09	–8.78	< 0.001
Depression	Stress	0.08	0.01	0.06	0.11	0.10	6.32	< 0.001
Depression	Age	–0.05	0.02	–0.08	–0.02	–0.03	–3.18	0.001
Depression	Physical health	0.52	0.07	0.38	0.66	0.07	7.30	< 0.001
Depression	Relationships	0.88	0.11	0.66	1.10	0.07	7.88	< 0.001
Depression	PTSD	0.14	0.01	0.12	0.15	0.27	15.24	< 0.001

Note. PTSD = posttraumatic stress disorder related to the COVID-19 pandemic, *CI* = confidence interval.

Table S3. Parameters estimates for path model in the Ukrainian sample (*n* = 1649)

Variables		*B*	*SE*	95% CI		β	*z*	*p*
**Dependent**	**Predictor**			**Lower**	**Upper**			
Life satisfaction	Physical health	–0.95	0.11	–1.14	–0.71	–0.15	–8.72	< 0.001
Life satisfaction	Anxiety	0.24	0.03	0.18	0.30	0.21	7.77	< 0.001
Life satisfaction	Depression	–0.24	0.03	–0.30	–0.20	–0.25	–9.37	< 0.001
Life satisfaction	Relationships	–1.35	0.18	–1.71	–1.01	v0.11	–7.63	< 0.001
Life satisfaction	Religiosity	0.56	0.10	0.35	0.76	0.08	5.45	< 0.001
Life satisfaction	Positive effects	0.18	0.02	0.15	0.21	0.19	11.26	< 0.001
Life satisfaction	Gender	1.11	0.18	0.75	1.48	0.09	6.23	< 0.001
Life satisfaction	Stress	–0.29	0.02	–0.33	–0.26	–0.35	–17.19	< 0.001
Life satisfaction	Age	0.08	0.03	0.02	0.13	0.03	2.73	0.006
Physical health	PA	–0.18	0.03	–0.24	–0.12	–0.09	–5.98	< 0.001
Physical health	Stress	0.02	0.00	0.02	0.03	0.19	9.02	< 0.001
Physical health	Age	0.02	0.00	0.01	0.03	0.05	4.82	< 0.001
Physical health	Anxiety	0.03	0.00	0.03	0.04	0.18	8.71	< 0.001
PTSD	Physical health	0.76	0.19	0.39	1.15	0.06	3.93	< 0.001
PTSD	Stress	0.38	0.03	0.32	0.44	0.22	13.03	< 0.001
PTSD	Anxiety	1.33	0.05	1.22	1.41	0.55	27.67	< 0.001
Anxiety	Stress	0.50	0.01	0.48	0.52	0.71	56.90	< 0.001
Depression	Anxiety	0.60	0.02	0.55	0.64	0.50	25.51	< 0.001
Depression	Religiosity	–0.58	0.07	–0.71	–0.45	–0.08	–8.78	< 0.001
Depression	Stress	0.08	0.01	0.06	0.11	0.10	6.32	< 0.001
Depression	Age	–0.05	0.02	–0.08	–0.02	–0.02	–3.18	0.001
Depression	Physical health	0.52	0.07	0.38	0.66	0.08	7.30	< 0.001
Depression	Relationships	0.88	0.11	0.66	1.10	0.07	7.88	< 0.001
Depression	PTSD	0.14	0.01	0.12	0.15	0.28	15.24	< 0.001

Note. PTSD = posttraumatic stress disorder related to the COVID-19 pandemic, *CI* = confidence interval.

Table S4. Estimation of path parameters for life satisfaction in Polish sample of university students (n = 1581)

Path parameter	*B*	*SE*	95% CI		β	*z*	*p*
			Lower	Upper			
PH ⇒ PTSD ⇒ Depression ⇒ LS	–0.03	0.01	–0.04	–0.01	0.00	–3.55	< 0.001
PH ⇒ Depression ⇒ LS	–0.13	0.02	–0.17	–0.09	–0.02	–5.95	< 0.001
Anxiety ⇒ PH ⇒ LS	–0.03	0.01	–0.04	–0.02	–0.03	–6.39	< 0.001
Anxiety ⇒ PH ⇒ PTSD ⇒ Depression ⇒ LS	0.00	0.00	0.00	0.00	0.00	–3.25	0.001
Anxiety ⇒ PH ⇒ Depression ⇒ LS	0.00	0.00	–0.01	0.00	0.00	–4.89	< 0.001
Anxiety ⇒ PTSD ⇒ Depression ⇒ LS	–0.05	0.01	–0.06	–0.04	–0.04	–7.74	< 0.001
Anxiety ⇒ Depression ⇒ LS	–0.15	0.02	–0.18	–0.11	–0.13	–8.60	< 0.001
RelStat1 ⇒ Depression ⇒ LS	–0.21	0.04	–0.29	–0.15	–0.02	–5.98	< 0.001
Religiosity ⇒ Depression ⇒ LS	0.14	0.02	0.10	0.19	0.02	6.29	< 0.001
Stress ⇒ PH ⇒ LS	–0.02	0.00	–0.03	–0.02	–0.03	–6.05	< 0.001
Stress ⇒ PH ⇒ PTSD ⇒ Depression ⇒ LS	0.00	0.00	0.00	0.00	0.00	–3.31	< 0.001
Stress ⇒ PH ⇒ Depression ⇒ LS	0.00	0.00	0.00	0.00	0.00	–4.98	< 0.001
Stress ⇒ PTSD ⇒ Depression ⇒ LS	–0.01	0.00	–0.02	–0.01	–0.02	–7.32	< 0.001
Stress ⇒ Anxiety ⇒ LS	0.12	0.02	0.09	0.15	0.15	7.69	< 0.001
Stress ⇒ Anxiety ⇒ PH ⇒ LS	-0.02	0.00	–0.02	–0.01	–0.02	–6.42	< 0.001
Stress ⇒ Anxiety ⇒ PH ⇒ PTSD ⇒ Depression ⇒ LS	0.00	0.00	0.00	0.00	0.00	–3.26	0.001
Stress ⇒ Anxiety ⇒ PH ⇒ Depression ⇒ LS	0.00	0.00	0.00	0.00	0.00	–4.89	< 0.001
Stress ⇒ Anxiety ⇒ PTSD ⇒ Depression ⇒ LS	–0.02	0.00	–0.03	–0.02	–0.03	–7.69	< 0.001
Stress ⇒ Anxiety ⇒ Depression ⇒ LS	–0.07	0.01	–0.09	–0.06	–0.09	–8.49	< 0.001
Stress ⇒ Depression ⇒ LS	–0.02	0.00	–0.03	–0.01	–0.03	–5.28	< 0.001
Age ⇒ PH ⇒ LS	–0.02	0.00	–0.03	-0.01	–0.01	–4.17	< 0.001
Age ⇒ PH ⇒ PTSD ⇒ Depression ⇒ LS	0.00	0.00	0.00	0.00	0.00	–2.75	0.006
Age ⇒ PH ⇒ Depression ⇒ LS	0.00	0.00	0.00	0.00	0.00	–3.69	< 0.001
Age ⇒ Depression ⇒ LS	0.01	0.00	0.01	0.02	0.01	2.97	0.003
PA ⇒ PH ⇒ LS	0.17	0.04	0.11	0.26	0.01	4.64	< 0.001
PA ⇒ PH ⇒ PTSD ⇒ Depression ⇒ LS	0.01	0.00	0.00	0.01	0.00	3.06	0.002
PA ⇒ PH ⇒ Depression ⇒ LS	0.02	0.01	0.01	0.04	0.00	4.18	< 0.001
PTSD ⇒ Depression ⇒ LS	–0.03	0.00	–0.04	–0.03	–0.07	–8.25	< 0.001

Table S5. Estimation of path parameters for life satisfaction in Ukrainian sample of university students (n = 1649)

Path parameter	B	SE	95% CI		β	z	p
			Lower	Upper			
PH ⇒ PTSD ⇒ Depression ⇒ LS	–0.03	0.01	–0.04	–0.01	0.00	–3.55	< 0.001
PH ⇒ Depression ⇒ LS	–0.13	0.02	–0.17	–0.09	–0.02	–5.95	< 0.001
Anxiety ⇒ PH ⇒ LS	–0.03	0.01	–0.04	–0.02	–0.03	–6.39	< 0.001
Anxiety ⇒ PH ⇒ PTSD ⇒ Depression ⇒ LS	0.00	0.00	0.00	0.00	0.00	–3.25	0.001
Anxiety ⇒ PH ⇒ Depression ⇒ LS	0.00	0.00	–0.01	0.00	0.00	–4.89	< 0.001
Anxiety ⇒ PTSD ⇒ Depression ⇒ LS	–0.05	0.01	–0.06	–0.04	–0.04	–7.74	< 0.001
Anxiety ⇒ Depression ⇒ LS	–0.15	0.02	–0.18	–0.11	–0.13	–8.60	< 0.001
RelStat1 ⇒ Depression ⇒ LS	–0.21	0.04	–0.29	–0.15	–0.02	–5.98	< 0.001
Religiosity ⇒ Depression ⇒ LS	0.14	0.02	0.10	0.19	0.02	6.29	< 0.001
Stress ⇒ PH ⇒ LS	–0.02	0.00	–0.03	–0.02	–0.03	–6.05	< 0.001
Stress ⇒ PH ⇒ PTSD ⇒ Depression ⇒ LS	0.00	0.00	0.00	0.00	0.00	–3.31	< 0.001
Stress ⇒ PH ⇒ Depression ⇒ LS	0.00	0.00	0.00	0.00	0.00	–4.98	< 0.001
Stress ⇒ PTSD ⇒ Depression ⇒ LS	–0.01	0.00	–0.02	–0.01	–0.02	–7.32	< 0.001
Stress ⇒ Anxiety ⇒ LS	0.12	0.02	0.09	0.15	0.15	7.69	< 0.001
Stress ⇒ Anxiety ⇒ PH ⇒ LS	–0.02	0.00	–0.02	–0.01	–0.02	–6.42	< 0.001
Stress ⇒ Anxiety ⇒ PH ⇒ PTSD ⇒ Depression ⇒ LS	0.00	0.00	0.00	0.00	0.00	–3.26	0.001
Stress ⇒ Anxiety ⇒ PH ⇒ Depression ⇒ LS	0.00	0.00	0.00	0.00	0.00	–4.89	< 0.001
Stress ⇒ Anxiety ⇒ PTSD ⇒ Depression ⇒ LS	–0.02	0.00	–0.03	–0.02	–0.03	–7.69	< 0.001
Stress ⇒ Anxiety ⇒ Depression ⇒ LS	–0.07	0.01	–0.09	–0.06	–0.09	–8.49	< 0.001
Stress ⇒ Depression ⇒ LS	–0.02	0.00	–0.03	–0.01	–0.02	–5.28	< 0.001
Age ⇒ PH ⇒ LS	–0.02	0.00	–0.03	–0.01	–0.01	–4.17	< 0.001
Age ⇒ PH ⇒ PTSD ⇒ Depression ⇒ LS	0.00	0.00	0.00	0.00	0.00	–2.75	0.006
Age ⇒ PH ⇒ Depression ⇒ LS	0.00	0.00	0.00	0.00	0.00	–3.69	< 0.001
Age ⇒ Depression ⇒ LS	0.01	0.00	0.01	0.02	0.01	2.97	0.001
PA ⇒ PH ⇒ LS	0.17	0.04	0.11	0.26	0.01	4.64	< 0.001
PA ⇒ PH ⇒ PTSD ⇒ Depression ⇒ LS	0.01	0.00	0.00	0.01	0.00	3.06	0.001
PA ⇒ PH ⇒ Depression ⇒ LS	0.02	0.01	0.01	0.04	0.00	4.18	< 0.001
PTSD ⇒ Depression ⇒ LS	–0.03	0.00	–0.04	–0.03	–0.07	–8.25	< 0.001
